# Supplementary material for: Patterns of Intron Gain and Loss in Fungi
Source: PLoS Biol. 2004 Nov 30;2(12):e422. doi: 10.1371/journal.pbio.0020422 (PMC532390; doi:10.1371/journal.pbio.0020422)
Supplement: Table S1 — Also available at http://genes.mit.edu/NielsenEtAl/. (4.3 MB ZIP). [file pbio.0020422.st001.zip › NielsenEtAl/html/801.html]

AN4073.1.NCU06432.1.MG06480.1.FG07292.1


```
 CLUSTAL W (1.82) Multiple Sequence Alignments - Introns Inserted


Sequence 1: NCU06432.1	132 aa
Sequence 2: FG07292.1	131 aa
Sequence 3: MG06480.1	121 aa
Sequence 4: AN4073.1	183 aa
Alignment Length: 183 aa
Number Identitical Residues: 99 aa
Alignment Score (without introns) 4219


MG06480.1 	-~------------------~----------MSVLDALKGVLKLALMHDGLARGLREASK
NCU06432.1	-~-----------------M0VSADAAPKGQMSVLDALKGVLKISLMHDGLARGLREASK
FG07292.1 	-~-----------------M0VSGD-APKGQMSILDALKGVLKLSLMHDGLARGLREASK
AN4073.1  	M0SDGEETQSNPPVAAEEVE~VPAESGAGGQMSVLDALKGVLRIALIHDGLARGLREAAK
          	  :....:.:... ::..    ...:.. ..**:********:::*:***********:*

MG06480.1 	ALDRRQAHMCVLNEACEEEAYKKLVIALCSEHKIPLIKVPDGKQLGEWAGLC1V------
NCU06432.1	ALDRREAHMCVLNESCEEEAYKKLVIALCSEHKIPLIKVPDGKQLGEWAGLC1V------
FG07292.1 	ALDRRQAHMCVLNENCEEEAYKKLVVALCNEHNIPLIQIPDGKQLGEWAGLC1V------
AN4073.1  	ALDRRQAHMCVLNEGCEEEAYKKLVVALCSEHKIPLIKVPDGKMLGEWVGLC~TYLEPPK
          	*****:******** **********:***.**:****::**** ****.*** .  ....

MG06480.1 	---------------------------LDREGNARKVVNCSCVVVKDWGEESQERS~VIL
NCU06432.1	---------------------------LDREGNARKVVNCSCVVVKNWGEMSSERE0ILL
FG07292.1 	---------------------------LDREGNARKVVNCSCVVVKDWGEESQERS~ILL
AN4073.1  	NNYQRTLEELQKFTIGRMLTVIYLTGQLDREGNARKVVNCSCVVVKDWGEESQERS~VLL
          	.. . : .. .. : .   :    :..*******************:*** *.**. ::*

MG06480.1 	NYFQTEQ
NCU06432.1	NYFQTEQ
FG07292.1 	NYFQTEQ
AN4073.1  	NYFQTEQ
          	*******
```
